# Investigation into Cellular Glycolysis for the Mechanism Study of Energy Metabolism Disorder Triggered by Lipopolysaccharide

**DOI:** 10.3390/toxins10110441

**Published:** 2018-10-29

**Authors:** Ruyuan Zhang, Jian Ji, Ivana Blaženović, Fuwei Pi, Tingwei Wang, Yinzhi Zhang, Xiulan Sun

**Affiliations:** 1 School of Food Science, State Key Laboratory of Food Science and Technology, National Engineering Research Center for Functional Foods, Jiangnan University, Wuxi 214122, China; zhangruyuanzry@hotmail.com (R.Z.); jijian@jiangnan.edu.cn (J.J.); pifuwei@jiangnan.edu.cn (F.P.); wangtingwei427@outlook.com (T.W.); yinzhizhang@jiangnan.edu.cn (Y.Z.); 2 Synergetic Innovation Center for Food Safety and Nutrition, Jiangnan University, Wuxi 214122, China; 3 NIHWest Coast Metabolomics Center, UC Davis Genome Center, University of California, Davis, CA 95616, USA; iblazenovic@ucdavis.edu

**Keywords:** lipopolysaccharide, energy metabolism, glycolysis, cancer

## Abstract

Lipopolysaccharide (LPS) is the main virulence factor of Gram-negative bacteria, which can incite inflammation in tissues by inducing cells to secrete a variety of proinflammatory mediators, including cytokines, chemokines, interleukins, and prostaglandins. Herein, we chose LPS as an inducer to establish an inflammatory model of HeLa cells, and explored the effects of LPS on energy metabolism. We treated HeLa cells with different concentrations (0, 0.4, 1.0, 2.0, 4.0, and 6.0 μg/mL) of LPS for 24 h, and explored its effects on intercellular adenosine triphosphate (ATP) levels, intercellular nitrous oxide (NO) content, mitochondrial functions, and enzyme activities related to energy metabolism. Furthermore, we used metabonomics to study the metabolites that participated in energy metabolism. We found a positive correlation between LPS concentrations and intracellular ATP levels. In addition, LPS increased intracellular NO production, altered mitochondrial functions, strengthened glycolytic enzyme activities, and changed metabolites related to energy metabolism. Hence, in this study, we showed that LPS can strengthen energy metabolism by enhancing glycolysis, which could be used as an early diagnostic biomarker or a novel therapeutic target for inflammation-associated cancers.

## 1. Introduction

Cancer is a worldwide public health problem and poses a severe threat to human health and life [1]. The tumor microenvironment (TME) plays a pivotal role in tumor initiation, progression, and metastasis. It is a complex and heterogeneous assembly characterized by elevated lactate levels, low glucose and nutrient levels, acidic extracellular pH levels, and multiple cytokines and growth factors [2,3]. Cancer cells have a strong demand for adenosine triphosphate (ATP) to sustain the anabolic processes of growth and proliferation. Furthermore, tumors prefer a hypoxic environment, utilizing glycolysis to produce ATP, even in the presence of sufficient oxygen [4,5], which is called the Warburg effect. Although metabolic reprogramming seems to decrease energy yields, this glycolytic phenotype increases glucose intake and aids glycolytic intermediates to proliferate efficiently. However, the Warburg effect can be weakened or strengthened when the tumor microenvironment conditions are changed [6,7,8].

Lipopolysaccharide (LPS), also named endotoxin, is an essential component of the outer membranes of Gram-negative (G^−^) bacteria [9]. The classical LPS molecule consists of lipid A, the core oligosaccharide, and the O antigen [10]. Lipid A is the center virulence and active site of LPS, which is composed of glucosamines, phosphates, and fatty acids [11]. As the main virulence factor of Gram-negative bacteria, LPS plays an important role in the development of Gram-negative bacterial infections and diseases, and is considered to be the primary cause of systemic inflammatory syndrome. It can activate toll-like receptors (TLRs) and nucleotide-binding oligomerization domain-like receptors (NLRs), in vivo and in vitro. For example, LPS is the main ligand for TLR-4, which can activate TLR-4 and initiate signal transduction and mediate natural immune and inflammatory reactions. Many studies confirmed that LPS could up-regulate different proinflammatory mediators including prostaglandin E2 (PGE2), nitric oxide species (NOS), and proinflammatory cytokines to induce inflammation [12,13]. It has been reported that pro-inflammatory cytokines in microenvironments were propitious for cancer progression and subsequent metastasis [14,15]. However, the metabolic manner of inflammation and transition process of cancer is unclear so far.

Cell metabolomics is a qualitative and quantitative analysis of a wide variety of intracellular small molecules, which plays an important role in studying the relationship between metabolites and physiological or pathological changes [16]. Metabolic pathways involved in energy metabolism mainly include glycolysis, the tricarboxylic acid (TCA) cycle, and the pentose phosphate pathway. Energy metabolic reprogramming is pivotal with cancer development [17,18]. There are plenty of studies that have investigated the relationship between inflammation and cancer [19,20], but few studies have focused on the effect of the inflammatory microenvironment on the energy metabolism of cancer cells. Metabolomics can be a useful means to study the reprogrammed energy metabolism of cancer cells in an inflammatory microenvironment.

In this work, we chose LPS as an inducer to establish an inflammatory model of HeLa cells, and explored the effects of LPS on energy metabolism. We treated HeLa cells with different concentrations (0, 0.4, 1.0, 2.0, 4.0, and 6.0 μg/mL) of LPS for 24 h, and explored its effects on intercellular ATP levels, intercellular nitrous oxide (NO) content, mitochondrial functions, and enzyme activities related to energy metabolism. Furthermore, we used metabonomics to study the metabolites that participated in energy metabolism. We showed that LPS can strengthen energy metabolism by enhancing glycolysis.

## 2. Results

### 2.1. The Effects of LPS on Intracellular ATP Levels

**Figure 1:**
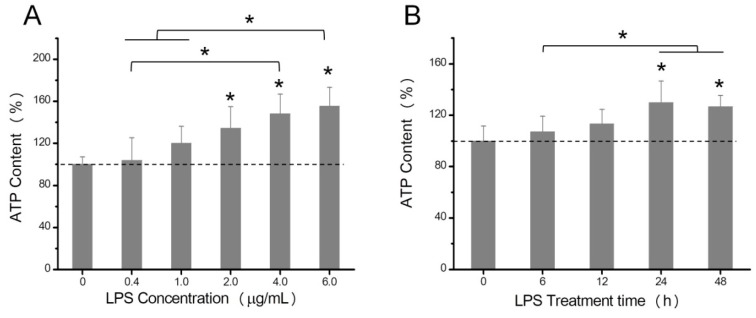
Effects of lipopolysaccharides (LPS) on the adenosine triphosphate (ATP) content in HeLa cells. HeLa cells were treated with different concentrations of LPS (0, 0.4, 1.0, 2.0, 4.0, and 6.0 µg/mL) for 24 h (**A**). HeLa cells were treated with 6 µg/mL LPS for different incubation times (0, 6, 12, 24, and 48 h) (**B**). The ATP content was expressed as a percentage of control values (0 µg/mL LPS). Data were presented as mean values with standard deviations. * *p* < 0.05.

As reported in many articles [21,22,23,24,25], after treatment with LPS, the content of TNF-α increased dramatically (Figure S1), which implied that the LPS stimulation could cause the inflammatory reaction in HeLa cells. The LPS-induced ATP concentration change was evaluated using the ATP assay kit. The luminescence intensity increased linearly with the increase in ATP concentration. As shown in Figure 1A, HeLa cells were treated with different concentrations of LPS (0, 0.4, 1.0, 2.0, 4.0, and 6.0 µg/mL) for 24 h. The ATP content of HeLa cells was positively correlated with LPS concentrations, especially when HeLa cells were stimulated with 6.0 µg/mL LPS, for which the intracellular ATP level rose by 55.4%. Figure 1B showed that the ATP levels increased first, and then they decreased with the increasing LPS treatment time, with maximum levels of ATP achieved at 24 h. The reason why intracellular ATP levels decreased in cells cultured for longer than 24 h was due to the consumption of nutrients, which influenced the cellular energy metabolism.

### 2.2. The Effects of LPS on NO Production, Mitochondrial ROS, and Mitochondrial Membrane Potential

**Figure 2:**
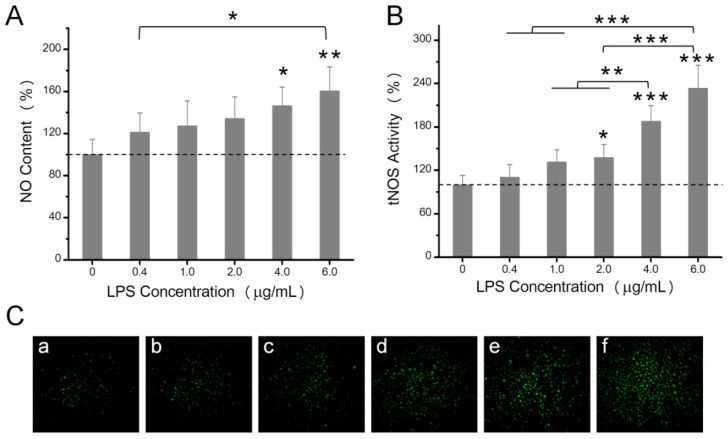
Effects of increasing concentrations of LPS (0, 0.4, 1.0, 2.0, 4.0, and 6.0 µg/mL) on NO content (**A**) and tNOS activity (**B**). Fluorescence image, acquired by high content analysis, showed the total tNOS activity in HeLa cells (**C**). Letters a, b, c, d, e, and f represented the LPS concentrations of 0, 0.4, 1.0, 2.0, 4.0, and 6.0 µg/mL, respectively. The NO content and tNOS activity were expressed as a percentage of control values (0 µg/mL LPS). Data were presented as mean values with standard deviations. * *p* < 0.05, ** *p* < 0.01, *** *p* < 0.001.

NO is the simplest signal molecule and has multiple physiological functions in organisms. As shown in Figure 2A, treating HeLa cells with increasing LPS concentrations (0.4, 1.0, 2.0, 4.0, and 6.0 µg/mL) for 24 h enhanced cellular NO content by 21.5%, 27.3%, 34.2%, 46.5%, and 60.4%, respectively. Meanwhile, NO synthase activity showed a positive correlation with the LPS treatment (Figure 2B,C).

**Figure 3:**
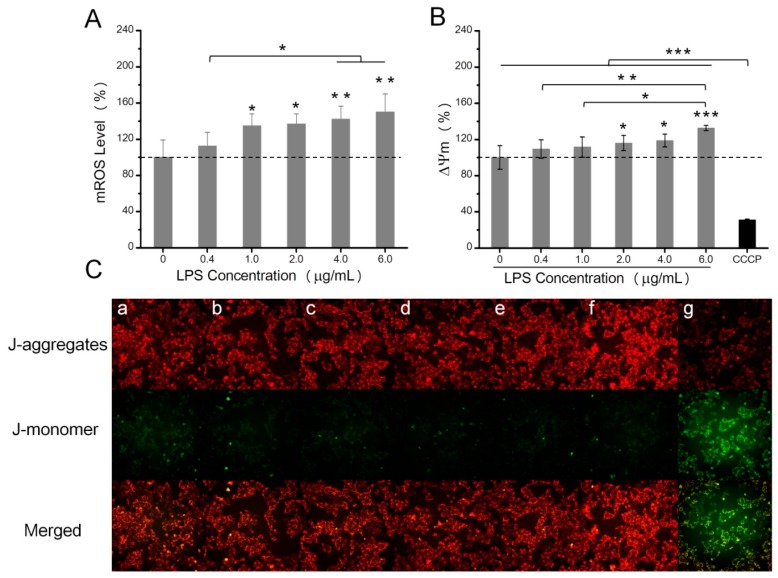
Effects of increasing concentrations of LPS (0, 0.4, 1.0, 2.0, 4.0, and 6.0 µg/mL) on mROS level (**A**) and ΔΨm (**B**,**C**) in HeLa cells. Fluorescence image acquired by high content analysis showed the ΔΨm level in HeLa cells (**C**). Letters a, b, c, d, e, f, and g represented the LPS concentrations of 0, 0.4, 1.0, 2.0, 4.0, 6.0 µg/mL, and carbonyl cyanide m-chlorophenylhydrazone (CCCP), respectively. The ΔΨm was expressed as a percentage of control values (0 µg/mL LPS). Data were presented as mean values with standard deviations. * *p* < 0.05, ** *p* < 0.01, *** *p* < 0.001.

The mitochondrion plays an important role in energy metabolism. Mitochondrial ROS and mitochondrial membrane potential are two important indexes of mitochondrial function. The mitochondrion was the major intracellular source of ROS [26], and the mitochondrial membrane potential provides transmembrane potential energy for the synthesis of ATP [27]. As shown in Figure 3A, with increased concentrations of LPS (0, 0.4, 1.0, 2.0, 4.0, and 6.0 µg/mL), the mitochondrial ROS level rose dramatically. Mitochondrial ROS increased by 50.3% in HeLa cells treated with 6.0 µg/mL LPS compared with the control.

The mitochondrial membrane potential of HeLa cells exhibited the same trend as the mitochondrial ROS after the treatment of different LPS concentrations (Figure 3B,C). The positive control, carbonyl cyanide m-chlorophenylhydrazone (CCCP), emitted weaker red fluorescence and stronger green fluorescence compared with the blank control, which implied a lower mitochondrial membrane potential of the positive control. After treatment with different concentrations of LPS, the ratio of red to green fluorescence intensity increased, which suggested a slight increase in the mitochondrial membrane potential.

### 2.3. The Effects of LPS on Enzyme Activities Related to Energy Metabolism

**Figure 4:**
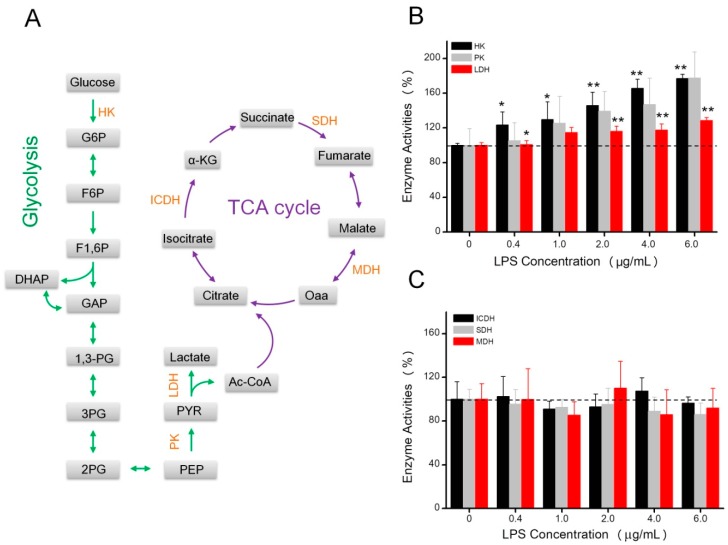
Effects of increasing concentrations of LPS (0, 0.4, 1.0, 2.0, 4.0, and 6.0 µg/mL) on enzyme activities involved in glycolysis and the tricarboxylic acid (TCA) cycle. (**A**) Showed the metabolism pathway of glycolysis and the TCA cycle. The activities of HK, PK, and LDH were shown in (**B**), and the activities of ICD, SDH, and MDH were shown in (**C**), expressed as a percentage of control values (0 µg/mL LPS). Data were presented as mean values with standard deviations. * *p* < 0.05, ** *p* < 0.01.

Several enzyme activities involved in glycolysis and the TCA cycle were determined. As shown in Figure 4B, after LPS treatments, the activities of the three glycolytic enzymes increased, which was most evident when cells were treated with 6 µg/mL LPS, since the activities of PK, HK, and LDH were increased 77.5%, 77.2%, and 28.7%, respectively. However, enzyme activities related to the TCA cycle, including ICDH, MDH, and SDH activities, exhibited no differences compared to controls (Figure 4C).

### 2.4. Analysis of Metabolites Related to Energy Metabolism

**Figure 5:**
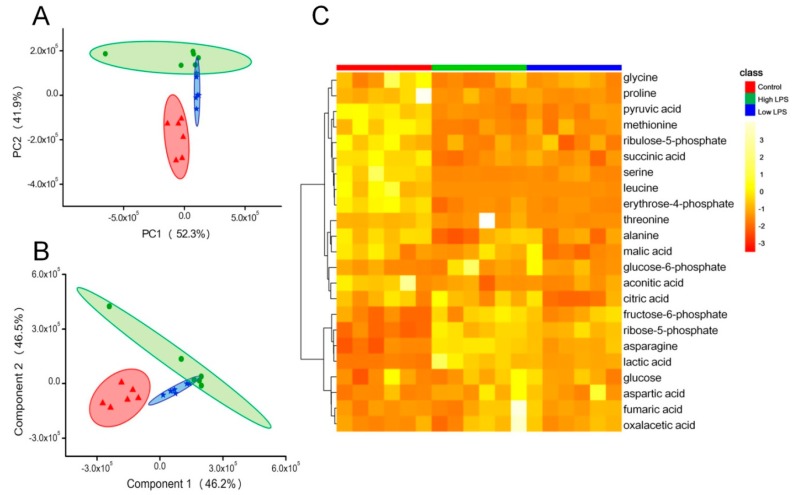
Metabolomic profiles of the three groups: The high LPS group (6.0 µg/mL, green), the low LPS group (0.4 µg/mL, blue), and the control group (red). In the PCA model, variance of PC1 was 52.3% and variance of PC2 was 41.9% (**A**). In the PLSDA model, variance of component 1 was 46.2% and variance of component 2 was 46.5% (**B**). (**C**) was the heatmap of the cluster analysis of the 23 metabolites related to energy metabolism.

For the sake of more clearly illustrating the effect of LPS on ATP generation, we applied metabonomics to study the metabolites in energy related metabolic pathways. The 23 metabolites related to energy metabolism were shown in Table S1. The results of PCA (Figure 5A) and PLSDA (Figure 5B) exhibited that metabolites in the LPS group and the control group were separated into different clusters. The high LPS group and the control group were far away from each other, and the low LPS group was in the middle. As shown in Figure 5C, the color of blocks in the heatmap changed from yellow to red, which indicated the corresponding up-regulation and down-regulation of the metabolites, respectively. Red, blue, and green represented the control group, the 0.4 µg/mL LPS treatment group, and the 6 µg/mL LPS treatment group, respectively. According to the distribution of colors, there was an obvious difference between the control group and the two LPS treatment groups. Both results indicated that stimulation with LPS indeed changed the energy metabolism of HeLa cells.

**Figure 6:**
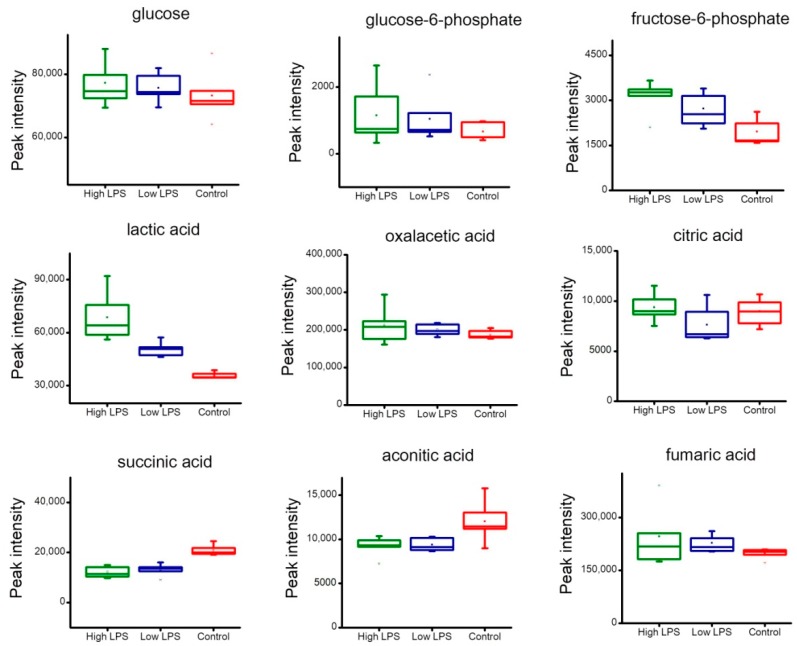
Boxplots of the peak intensity change of metabolites related to glycolysis and the TCA cycle. The high LPS group (6.0 µg/mL, green), the low LPS group (0.4 µg/mL, blue), and the control group (red).

**Figure 7:**
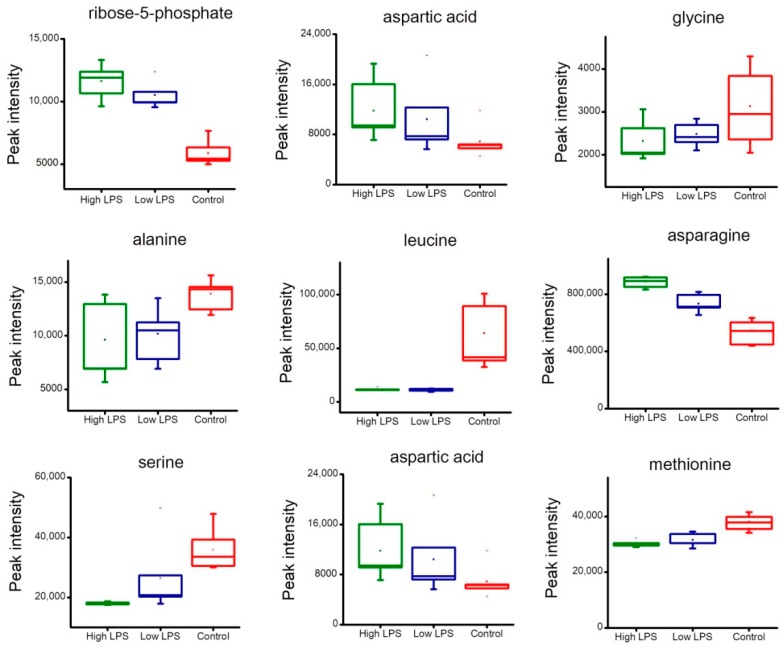
Boxplots of the peak intensity change of ribose-5-phosphate and amino acid. The high LPS group (6.0 µg/mL, green), the low LPS group (0.4 µg/mL, blue), and the control group (red).

Figure 6 showed the peak intensity change of metabolites related to energy metabolism. After the stimulation with LPS, glucose level had little change, glucose-6-phosphate level had a small increase, and fructose-6-phosphate level had an obvious increase. Lactic acid was the final product of glycolysis, which up-regulated dramatically with the treatment of LPS. The metabolites involved in the TCA cycle had no obvious difference between the LPS treatment group and the control group. In Figure 7, ribose-5-phosphate level rose obviously with the stimulation of LPS. The content of serine, methionine, leucine, aspartic acid, and asparagine changed dramatically, which implied that the LPS treatment dramatically altered the amino acid metabolism.

## 3. Discussion

Cancer development related to the alteration of cell metabolism and reprogrammed energy metabolism is an emerging hallmark of cancer cells [28]. In this work, we evaluated the effects of LPS on the energy metabolism of HeLa cells. After LPS stimulation, ATP levels in HeLa cells increased, which indicated changes in energy metabolism. NO plays an important role in the occurrence and development of energy metabolism-related diseases [29,30,31]. Furthermore, recently NO was shown to enhance glycolysis [32,33]. In our study, LPS increased NO release and NOS activity that promoted glycolysis.

The mitochondrion is essential to energy metabolism. Increased mitochondrial ROS levels and mitochondrial membrane potentials suggested that changes in mitochondrial function occurred. It is convincing that a low-level increase in ROS strengthens the signaling pathways to activate NFκB for cell survival, hypoxia-inducible factors (HIFs) for metabolic adaptation, and mitogen-activated protein (MAP) kinases for cell proliferation [34]. The mitochondrial membrane potential was thought to correlate with tumorigenicity, malignancy, and cell differentiation status [35]. It has been reported that cells with high mitochondrial membrane potentials had stronger resistance to the inducers of apoptosis compared with cells that had low mitochondrial membrane potentials [36]. Hence, variations in mitochondrial ROS and mitochondrial membrane potentials implied that a functional change in mitochondria occurred.

Glycolysis is the pathway of utilizing glucose to generate pyruvate or lactate, and not only produces two ATP molecules but also provides substrates for lipogenesis and glycogenesis storage pathways (Figure 4A). Hexokinase (HK) catalyzes the phosphorylation of glucose into glucose-6-phosphate and is the first enzyme, and one of the three rate-limiting enzymes, that participates in the glycolytic pathway. It has been reported that cancer cells have higher HK activity to accelerate the glycolytic process [37,38,39]. Pyruvate kinase (PK) is the enzyme that catalyzes the conversion of phosphoenolpyruvate to pyruvate. Increased PK activity results in cell metabolic changes that enable cells to proliferate under limited nutrient supplies [40]. Lactate dehydrogenase (LDH) plays a key role in regulating the conversion of glycolysis aerobic oxidation. LDH can catalyze pyruvate, a product of glycolysis, to lactate, which can acidify microenvironments to favor survival and growth of cancer cells in the hypoxic environment [41]. In this study, after stimulation by different LPS concentrations, HK, PK, and LDH activities were all increased, suggesting that the glycolytic pathway was enhanced. Meanwhile, the accumulation of fructose-6-phosphate and lactic acid in HeLa cells provided further evidence of the strengthened glycolysis.

The tricarboxylic acid (TCA) cycle is a core pathway in the metabolic processes of sugars, amino acids, and lipids [42]. The TCA cycle usually oxidizes the acetyl moiety of acetyl-CoA to CO_2_ in a cyclic mitochondrial route (Figure 4A). During the TCA cycle, NADPH and FADH2 can be generated to provide electrons to the mitochondrial respiratory chain for ATP production. Succinic dehydrogenase (SDH) is an important mitochondrial enzyme that catalyzes succinate oxidation in the TCA cycle, and couples transmission of electrons to ubiquinone in the respiratory chain [43]. It has been reported that the loss function of mitochondrial SDH contributed to hereditary tumors and the accumulation of succinate-activated HIF and its downstream glycolytic pathway [44,45,46]. MDH is also an essential enzyme of the TCA cycle with a major role in catalyzing the oxidation of malate to oxaloacetate. IDCH can oxidize isocitrate to α-ketoglutarate, with the reduction of NAD(P)^+^ to NAD(P)H, and plays a central role in the TCA cycle [47,48]. However, SDH, MDH, and ICDH activities exhibited no differences compared to controls. Meanwhile, the metabolites involved in the TCA cycle had no obvious difference between the LPS treatment group and the control group, which indicated that LPS stimulation had negligible impact on the TCA cycle.

Figure 7 exhibited the levels of ribose-5-phosphate and the amino acid change in HeLa cells. Ribose-5-phosphate was an essential precursor of nucleotides and its up-regulation suggested vigorous nucleic acid synthesis. Amino acid metabolism is closely related to glycolysis and the TCA cycle. Amino acid can provide a complementary substance for glycolysis and the TCA cycle. The content change of amino acid also reflected the reprogramming of energy metabolism.

The relationship between inflammation and metabolism has become a hot research topic for many years [49,50]. It has been confirmed that M1 macrophage tend to be much more dependent on glycolysis [51], while M2 macrophage prefer oxidative phosphorylation to acquire energy [52,53]. Some transcription factors, such as hypoxia-inducible factor-1α (HIF-1α), play important roles in metabolic reprogramming [54]. The peroxisome proliferator-activated receptor (PPAR-γ) is also an important immune–metabolism switch transcription factor. It has been reported that abscisic acid could regulate inflammation via ligand-binding domain-independent activation of PPAR-γ [55]. In our research, we found that after the LPS stimulation, HeLa cells exhibited strengthened glycolysis. Dingding Qu et al. have demonstrated that inflammation up-regulated the expression of key glycolytic enzymes via activation of the STAT3–c-Myc signaling pathway in colorectal cancer cells [56]. It is very interesting to study the underlying mechanisms of LPS induced metabolic reprogramming and how transcription factors work after LPS treatment in HeLa cells.

## 4. Conclusions

In this work, we found that LPS could increase the ATP level in HeLa cells. Preliminarily, we showed that the underlying mechanisms of the ATP concentration increased, and found that the increased ATP was generated from the strengthened glycolysis rather than the TCA cycle. LPS stimulation up-regulated NO levels, changed mitochondrial functions, strengthened glycolytic enzyme activities, and changed metabolites related to energy metabolism. The metabolic alterations were related to the character of the Warburg effect. Perhaps, this feature of metabolism reprogramming could be used as an early diagnostic biomarker or a novel therapeutic target for inflammation-associated cancers.

## 5. Materials and Methods

### 5.1. Chemicals and Reagents

Lipopolysaccharide (LPS, from *Escherichia coli* O111:B4, purified by gel-filtration chromatography), methoxyamine hydrochloride, N-Methyl-N-(trimethylsilyl) trifluoroacetamide (MSTFA), and fatty acid methyl esters (FAMEs) were purchased from Sigma-Aldrich (St. Louis, MO, USA). The reagents not mentioned above were all HPLC grade. Ultrapure water was purified with the Ultrapure Water Purification Systems (Millipore, Billerica, MA, USA).

### 5.2. Cell Culture and Treatment

HeLa cells were obtained from the Cell Bank of the Chinese Academy of Sciences (Shanghai, China). HeLa cells were cultured in Dulbecco’s Modification of Eagle Medium (DMEM) containing 10% heat-inactivated fetal bovine serum (FBS) at 37 °C in a 5% CO_2_ incubator. HeLa cells were treated with different concentrations of LPS (0, 0.4, 1.0, 2.0, 4.0, and 6.0 µg/mL) for 24 h. All cells used for the experiments were in the logarithmic phase of growth.

### 5.3. ATP Measurements

ATP levels in HeLa cells were measured using the ATP assay kit (Beyotime, China) based on firefly luciferase following the manufacturer’s instructions. In brief, HeLa cells were seeded into 6-well plates at the density of 4 × 10^5^ cells/well and treated with different concentrations of LPS (0, 0.4, 1.0, 2.0, 4.0, and 6.0 µg/mL). After 24 h, the cells were lysed and centrifuged. The ATP levels in the supernatants were determined using the assay buffers and the luminance was measured using a Microplate Reader (M5, Molecular Devices, San Francisco, CA, USA).

### 5.4. Nitrite Oxide Measurements

The NO assay kit (S0023, Biyuntian, China) was applied to measure total nitrites. After different LPS concentration treatments (0, 0.4, 1.0, 2.0, 4.0, and 6.0 µg/mL) for 24 h, the cells were lysed, collected, and transferred into 96-well plates with 50 μL of culture supernatant. Then, the Griess Reagent I and Griess Reagent II were loaded according to the manufacturer’s instructions. The absorbance at 540 nm was measured after incubation at room temperature for 10 min.

Total nitric oxide synthase (tNOS) activity was measured using the nitric oxide synthase assay kit (S0025, Biyuntian, China). HeLa cells were seeded into 96-well plates and stimulated with LPS for 24 h. Then, a fluorescent probe (DAF-FM DA) was added and incubated at 37 °C for 30 min in the dark. The high content screening microscope (ImageXpress Micro XLS, Molecular Devices, USA) was employed for fluorescence microscopy imaging at the excitation and emission wavelengths of 495 and 515 nm, respectively.

### 5.5. Mitochondrial ROS Detection

Mitochondrial ROS production was measured using the MitoSOX™ Red mitochondrial superoxide indicator. This novel fluorogenic dye is highly specific for superoxide recognition in the mitochondria of live cells. HeLa cells were seeded in 96-well plates and stimulated with different doses (0, 0.4, 1.0, 2.0, 4.0, and 6.0 µg/mL) of LPS for 24 h. After a brief wash, 100 μL of 5 μM MitoSOX™ reagent working solution was applied to cover the HeLa cells that adhered to coverslips, and the cells were incubated for 10 min at 37 °C in the dark. Next, HeLa cells were washed gently with warm PBS three times. Mitochondrial ROS measured using a Microplate Reader (M5, Molecular Devices, USA) with an excitation wavelength of 510 nm and a maximum emission wavelength of 580 nm.

### 5.6. Mitochondrial Membrane Potential Assessment

A JC-1 probe was used to determine the mitochondrial membrane potential (ΔΨm). It is a cationic dye that shows potential-dependent aggregation in mitochondria and exhibits a fluorescence change from green to red. Briefly, cells were seeded into 96-well plates and incubated in a 5% CO_2_ incubator at 37 °C for 12 h. After treatment with LPS, the cells were stained with JC-1 following the instructions of the mitochondrial membrane potential assay kit (C2006, Beyotime, China). The mitochondrial membrane potential was determined by the fluorescence intensity ratio of red to green. Carbonyl cyanide m-chlorophenylhydrazone (CCCP), which inhibits mitochondrial electron transport, was used as a positive control.

### 5.7. Determination of Enzyme Activities Related to Energy Metabolisms

LDH, PK, and HK are important enzymes during glycolysis. SDH, ICDH, and MDH are the important enzymes during the TCA cycle. The above six enzyme activities were determined using the enzyme activity test kit from Comin Biotechnology Co. Ltd. (Suzhou, China). HeLa cells were seeded into 6-well plates and stimulated with different concentrations of LPS (0, 0.4, 1.0, 2.0, 4.0, and 6.0 µg/mL) for 24 h. For each sample, 1 × 10^7^ cells were collected, lysed, and centrifuged, and measurements were performed following the manufacturer’s instructions.

### 5.8. Metabolite Extraction

Cell dishes were placed on the ice and washed twice with 1 mL chilled water. Each dish had 1 mL of quenching solvent (ice-cold methanol/H2O (3:2)) added to quench the media sample. Cells were manually scraped and centrifuged (1000× *g*, 5 min), then the supernatant was discarded and the precipitate was either stored at −80 °C or analyzed immediately. One mL of extraction solvent (acetonitrile/isopropanol/H_2_O (3:3:2, *v*/*v*)) was added to each tube and ground for 1500 rpm, 30 s, 5 cycles using a GenGrinder. Following centrifugation, 950 μL supernatant was collected and divided into two aliquots; one for analysis and the other as backup. A mixture from 10 μL of each sample was used as quality control.

### 5.9. Derivatization

10 μL of the *O*-methylhydroxylamine reagent solution (40 mg/mL) was mixed with dried samples and shaken for 90 min at 30 °C. Then *N*-methyl-*N* trimethylsilyltrifluoroacetamide (MSTFA) with 1% trimethylchlorosilane (TMCS) were applied to improve volatility of metabolites. Standard mixture of fatty acid methyl esters (FAMEs, C8–C16: 1 mg/mL; C18–C24: 0.5 mg/mL in chloroform) of 10 μL was added to the sample.

### 5.10. GC-TOF/MS Analysis

An Agilent 7890 gas chromatograph system coupled with a Pegasus HT time-of-flight mass spectrometer was used to perform the GC-TOF/MS analysis. The system used a Rxi-5Sil MS column (30 m × 250 mm inner diameter, 0.25 mm film thickness; Restek, Bellefonte, PA, USA). The sample volume was 1 μL and the carrier gas was helium. The front inlet purge flow and the column gas flow rate was 5 mL/min^−1^ and 20 mL/min^−1^, respectively. The initial temperature was maintained at 50 °C for 0.5 min, subsequently raised to 320 °C at a rate of 15 °C/min^−1^, and was kept for 8 min at 320 °C. The temperatures of injection, transfer line, and ion source were 320, 320, and 230 °C, respectively. The EI voltage was −70 eV in electron impact mode.

## Supplementary Materials

The following are available online at http://www.mdpi.com/2072-6651/10/11/441/s1, Figure S1: Effects of different concentrations of LPS (0, 0.4, 1.0, 2.0, 4.0, and 6.0 µg/mL) on TNF-αcontent (**A**). Effects of different concentrations of LPS (0, 0.4, 1.0, 2.0, 4.0, and 6.0 µg/mL) on IL-6 content (**B**). The content was expressed as a percentage of control values (0 µg/mL LPS). Data were presented as mean values with standard deviations. * *p* <0.05, ** *p* <0.01, *** *p* <0.001, Table S1: List of the 23 metabolites related to energy metabolism.

## References

[1] Favoriti P., Carbone G., Greco M., Pirozzi F., Pirozzi R.E., Corcione F. (2016). Worldwide burden of colorectal cancer: A review. Updat. Surg..

[2] Andersen A.P., Moreira J.M., Pedersen S.F. (2014). Interactions of ion transporters and channels with cancer cell metabolism and the tumour microenvironment. Philso. Trans. R. Soc. Lond..

[3] Pedersen A.K., Melo J.M.L.D., Mørup N., Tritsaris K., Pedersen S.F. (2017). Tumor microenvironment conditions alter Akt and Na^+^/H^+^ exchanger NHE1 expression in endothelial cells more than hypoxia alone: Implications for endothelial cell function in cancer. BMC Cancer.

[4] Airley R.E., Mobasheri A. (2007). Hypoxic regulation of glucose transport, anaerobic metabolism and angiogenesis in cancer: Novel pathways and targets for anticancer therapeutics. Chemotherapy.

[5] Warburg O. (1956). On the Origin of Cancer Cells. Science.

[6] Birsoy K., Possemato R., Lorbeer F.K., Bayraktar E.C., Thiru P., Yucel B., Wang T., Chen W.W., Clish C.B., Sabatini D.M. (2014). Metabolic determinants of cancer cell sensitivity to glucose limitation and biguanides. Nature.

[7] Corbet C., Pinto A., Martherus R., Jp S.D.J., Polet F., Feron O. (2016). Acidosis Drives the Reprogramming of Fatty Acid Metabolism in Cancer Cells through Changes in Mitochondrial and Histone Acetylation. Cell Metab..

[8] DeNicola G.M., Cantley L.C. (2015). Cancer’s Fuel Choice: New Flavors for a Picky Eater. Mol. Cell.

[9] Rietschel E.T., Brade H., Holst O., Brade L., Müller-Loennies S., Mamat U., Zähringer U., Beckmann F., Seydel U., Brandenburg K. (1996). Bacterial Endotoxin: Chemical Constitution, Biological Recognition, Host Response, and Immunological Detoxification.

[10] Caroff M., Karibian D. (2003). Structure of bacterial lipopolysaccharides. Carbohydr. Res..

[11] Needham B.D., Trent M.S. (2013). Fortifying the barrier: The impact of lipid A remodelling on bacterial pathogenesis. Nat. Rev. Microbiol..

[12] Badshah H., Ali T., Rehman S.U., Amin F.U., Ullah F., Kim T.H., Kim M.O. (2016). Protective Effect of Lupeol Against Lipopolysaccharide-Induced Neuroinflammation via the p38/c-Jun N-Terminal Kinase Pathway in the Adult Mouse Brain. J. Neuroimmune Pharmacol..

[13] Parajuli B., Sonobe Y., Kawanokuchi J., Doi Y., Noda M., Takeuchi H., Mizuno T., Suzumura A. (2012). GM-CSF increases LPS-induced production of proinflammatory mediators via upregulation of TLR4 and CD14 in murine microglia. J. Neuroinflammation.

[14] Chiang A.C., Massagué J. (2005). Molecular basis of metastasis. N. Engl. J. Med..

[15] Mantovani A., Allavena P., Sica A., Balkwill F. (2008). Cancer-related inflammation. Nature.

[16] Beger R.D., Dunn W., Schmidt M.A., Gross S.S., Kirwan J.A., Cascante M., Brennan L., Wishart D.S., Oresic M., Hankemeier T. (2016). Metabolomics enables precision medicine: “A White Paper, Community Perspective”. Metabol. Off. J. Metabol. Soc..

[17] Kim S.Y. (2018). Cancer Energy Metabolism: Shutting Power *off* Cancer Factory. Biomol. Ther..

[18] Snezhkina A.V., Krasnov G.S., Zaretsky A.R., Zhavoronkov A., Nyushko K.M., Moskalev A.A., Karpova I.Y., Afremova A.I., Lipatova A.V., Kochetkov D.V. (2016). Differential expression of alternatively spliced transcripts related to energy metabolism in colorectal cancer. BMC Genom..

[19] Korniluk A., Koper O., Kemona H., Dymickapiekarska V. (2017). From inflammation to cancer. Ir. J. Med. Sci..

[20] Pesic M., Greten F.R. (2016). Inflammation and cancer: Tissue regeneration gone awry. Curr. Opin. Cell Biol..

[21] Wang X.H., Yan G.T., Wang L.H., Hao X.H., Zhang K., Xue H. (2004). The mediating role of cPLA2 in IL-1 beta and IL-6 release in LPS-induced HeLa cells. Cell Biochem. Funct..

[22] Wang J., Lin D., Peng H., Shao J., Gu J. (2014). Cancer-derived immunoglobulin G promotes LPS-induced proinflammatory cytokine production via binding to TLR4 in cervical cancer cells. Oncotarget.

[23] Mineshiba J., Myokai F.F., Matsuura K., Nishimura F., Takashiba S. (2005). Transcriptional regulation of beta-defensin-2 by lipopolysaccharide in cultured human cervical carcinoma (HeLa) cells. Pathog. Dis..

[24] Lin M.C., Pan C.Y., Hui C.F., Chen J.Y., Wu J.L. (2013). Shrimp anti-lipopolysaccharide factor (SALF), an antimicrobial peptide, inhibits proinflammatory cytokine expressions through the MAPK and NF-κB pathways in LPS-induced HeLa cells. Peptides.

[25] Cheng Y.X., Qi X.Y., Huang J.L., Hu M., Zhou L.M., Li B.S., Xu X.X. (2012). Toll-like receptor 4 signaling promotes the immunosuppressive cytokine production of human cervical cancer. Eur. J. Gynaecol. Oncol..

[26] Shimura T., Sasatani M., Kamiya K., Kawai H., Inaba Y., Kunugita N. (2016). Mitochondrial reactive oxygen species perturb AKT/cyclin D1 cell cycle signaling via oxidative inactivation of PP2A in lowdose irradiated human fibroblasts. Oncotarget.

[27] Martínezreyes I., Diebold L.P., Kong H., Schieber M., Huang H., Hensley C.T., Mehta M.M., Wang T., Santos J.H., Woychik R. (2016). TCA Cycle and Mitochondrial Membrane Potential Are Necessary for Diverse Biological Functions. Mol. Cell.

[28] Hanahan D., Weinberg R. (2011). Hallmarks of Cancer: The Next Generation. Cell.

[29] Fetterman J.L., Holbrook M., Flint N., Feng B., Bretónromero R., Linder E.A., Berk B.D., Duess M.A., Farb M.G., Gokce N. (2016). Restoration of autophagy in endothelial cells from patients with diabetes mellitus improves nitric oxide signaling. Atherosclerosis.

[30] Kraehling J.R., Sessa W.C. (2017). Contemporary Approaches to Modulating the Nitric Oxide-cGMP Pathway in Cardiovascular Disease. Circ. Res..

[31] Kulaksızoglu S., Karalezli A. (2016). Aqueous Humour and Serum Levels of Nitric Oxide, Malondialdehyde and Total Antioxidant Status in Patients with Type 2 Diabetes with Proliferative Diabetic Retinopathy and Nondiabetic Senile Cataracts. Can. J. Diabetes.

[32] Brix B., Mesters J.R., Pellerin L., Jöhren O. (2012). Endothelial cell-derived nitric oxide enhances aerobic glycolysis in astrocytes via HIF-1α-mediated target gene activation. J. Neurosci. Off. J. Soc. Neurosci..

[33] Li L., Zhu L., Hao B., Gao W., Wang Q., Li K., Wang M., Huang M., Liu Z., Yang Q. (2017). iNOS-derived nitric oxide promotes glycolysis by inducing pyruvate kinase M2 nuclear translocation in ovarian cancer. Oncotarget.

[34] Sena L., Chandel N. (2012). Physiological Roles of Mitochondrial Reactive Oxygen Species. Mol. Cell.

[35] Ye X.Q., Li Q., Wang G.H., Sun F.F., Huang G.J., Bian X.W., Yu S.C., Qian G.S. (2011). Mitochondrial and energy metabolism-related properties as novel indicators of lung cancer stem cells. Int. J. Cancer.

[36] Pietilä M., Lehtonen S., Närhi M., Hassinen I.E., Leskelä H.V., Aranko K., Nordström K., Vepsäläinen A., Lehenkari P. (2010). Mitochondrial function determines the viability and osteogenic potency of human mesenchymal stem cells. Tissue Eng. Part C Methods.

[37] Altenberg B., Greulich K.O. (2004). Genes of glycolysis are ubiquitously overexpressed in 24 cancer classes. Genomics.

[38] Neary C.L., Pastorino J.G. (2010). Nucleocytoplasmic shuttling of hexokinase II in a cancer cell. Biochem. Biophys. Res. Commun..

[39] Palmieri D., Fitzgerald D., Shreeve S.M., Hua E., Bronder J.L., Weil R.J., Davis S., Stark A.M., Merino M.J., Kurek R. (2009). Analyses of Resected Human Brain Metastases of Breast Cancer Reveal the Association between Up-regulation of Hexokinase 2 and Poor Prognosis. Mol. Cancer Res..

[40] Benesch C., Schneider C., Voelker H.U., Kapp M., Caffier H., Krockenberger M., Dietl J., Kammerer U., Schmidt M. (2010). The clinicopathological and prognostic relevance of pyruvate kinase M2 and pAkt expression in breast cancer. Anticancer Res..

[41] Shuch B., Linehan W.M., Srinivasan R. (2013). Aerobic glycolysis: A novel target in kidney cancer. Expert Rev. Anticancer Ther..

[42] Scheffler I.E. (2007). Mitochondria.

[43] Nelson D.L., Cox M.M. (2008). Lehninger Principles of Biochemistry.

[44] King A., Selak M.A., Gottlieb E. (2006). Succinate dehydrogenase and fumarate hydratase: Linking mitochondrial dysfunction and cancer. Oncogene.

[45] Pollard P.J., Briã¨Re J.J., Alam N.A., Barwell J., Barclay E., Wortham N.C., Hunt T., Mitchell M., Olpin S., Moat S.J. (2005). Accumulation of Krebs cycle intermediates and over-expression of HIF1alpha in tumours which result from germline FH and SDH mutations. Hum. Mol. Gen..

[46] Tseng P.L., Wu W.H., Hu T.H., Chen C.W., Cheng H.C., Li C.F., Tsai W.H., Tsai H.J., Hsieh M.C., Chuang J.H. (2018). Decreased succinate dehydrogenase B in human hepatocellular carcinoma accelerates tumor malignancy by inducing the Warburg effect. Sci. Rep..

[47] Han S.J., Jang H.S., Noh M.R., Kim J., Kong M.J., Kim J.I., Park J.W., Park K.M. (2016). Mitochondrial NADP+-Dependent Isocitrate Dehydrogenase Deficiency Exacerbates Mitochondrial and Cell Damage after Kidney Ischemia-Reperfusion Injury. J. Am. Soc. Nephrol. Jasn.

[48] Reitman Z.J., Yan H. (2010). Isocitrate Dehydrogenase 1 and 2 Mutations in Cancer: Alterations at a Crossroads of Cellular Metabolism. J. Natl. Cancer Inst..

[49] Tawakol A., Singh P., Mojena M., Pimentel-Santillana M., Emami H., Macnabb M., Rudd J.H., Narula J., Enriquez J.A., Través P.G. (2015). HIF-1Î± and PFKFB3 Mediate a Tight Relationship Between Proinflammatory Activation and Anerobic Metabolism in Atherosclerotic Macrophages. Arterioscler. Thromb. Vasc. Biol..

[50] Haschemi A., Kosma P., Gille L., Evans C.R., Burant C.F., Starkl P., Knapp B., Haas R., Schmid J.A., Jandl C. (2012). The Sedoheptulose Kinase CARKL Directs Macrophage Polarization through Control of Glucose Metabolism. Cell Metab..

[51] Blagih J., Jones R. (2012). Polarizing Macrophages through Reprogramming of Glucose Metabolism. Cell Metab..

[52] Van den Bossche J., Baardman J., Otto N.A., Van der Velden S., Neele A.E., Sm V.D.B., Luque-Martin R., Chen H.J., Boshuizen M.C., Ahmed M. (2016). Mitochondrial Dysfunction Prevents Repolarization of Inflammatory Macrophages. Cell Rep..

[53] Tan Z., Xie N., Cui H., Moellering D.R., Abraham E., Thannickal V.J., Liu G. (2015). Pyruvate dehydrogenase kinase 1 participates in macrophage polarization via regulating glucose metabolism. J. Immunol..

[54] Kelly B., O’Neill L.A. (2015). Metabolic reprogramming in macrophages and dendritic cells in innate immunity. Cell Res..

[55] Bassaganyariera J., Guri A.J., Lu P., Climent M., Carbo A., Sobral B.W., Horne W.T., Lewis S.N., Bevan D.R., Hontecillas R. (2011). Abscisic Acid Regulates Inflammation via Ligand-binding Domain-independent Activation of Peroxisome Proliferator-activated Receptor γ. J. Biol. Chem..

[56] Qu D., Shen L., Liu S., Li H., Ma Y., Zhang R., Wu K., Yao L., Li J., Zhang J. (2017). Chronic inflammation confers to the metabolic reprogramming associated with tumorigenesis of colorectal cancer. Cancer Biol. Ther..

