# Supplementary material for: Investigation into Cellular Glycolysis for the Mechanism Study of Energy Metabolism Disorder Triggered by Lipopolysaccharide
Source: Toxins (Basel). 2018 Oct 29;10(11):441. doi: 10.3390/toxins10110441 (PMC6266602; doi:10.3390/toxins10110441)
Supplement: Supplementary file 1 [file toxins-10-00441-s001.pdf]

Supplementary Materials: Investigation into Cellular Glycolysis for Mechanism Study of Energy Metabolism Disorder Triggered by Lipopolysaccharide

Ruyuan Zhang, Jian Ji, Ivana Blaženović, Fuwei Pi, Tingwei Wang, Yinzhi Zhang and Xiulan Sun

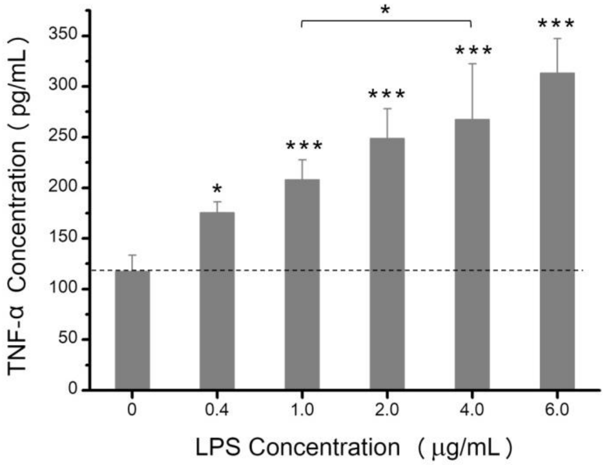

Figure S1. Effects of different concentrations of LPS (0, 0.4, 1.0, 2.0, 4.0, 6.0 μg/mL) on TNF-α content measured by ELISA. Data were presented as mean values with standard deviations. * $p < 0.05$, ** p < 0.01, *** p < 0.001.

Table S1. List of the 23 metabolites related to energy metabolism.

Number	t_R (min)	Nme	*m*/*z*	Relative Intensity High LPS	LOW LPS	Control
1	5.4	pyruvic acid	248	4.17×10^4	4.74×10^4	1.24×10^5
2	6.14	alanine	184	2.57×10^4	2.72×10^4	3.71×10^4
3	7.71	leucine	243	3.13×10^4	3.00×10^4	1.70×10^5
4	7.91	glycine	285	6.18×10^3	6.63×10^3	8.36×10^3
5	8.22	succinic acid	85	3.27×10^4	3.51×10^4	5.57×10^4
6	8.43	proline	85	3.96×10^4	3.75×10^4	7.06×10^4
7	8.58	serine	217	4.80×10^4	5.18×10^4	9.55×10^4
8	8.76	threonine	103	7.76×10^5	3.68×10^5	7.95×10^5
9	8.85	fumaric acid	149	6.55×10^5	6.07×10^5	5.30×10^5
10	9.5	oxalacetic acid	149	5.69×10^5	5.36×10^5	5.00×10^5
11	9.52	aspartic acid	174	3.15×10^4	2.78×10^4	1.83×10^4
12	9.64	malic acid	217	3.03×10^4	2.51×10^4	3.38×10^4
13	10.23	methionine	85	8.03×10^4	8.41×10^4	1.02×10^5
14	11.16	asparagine	117	2.37×10^6	1.95×10^6	1.45×10^6
15	11.78	aconitic acid	98	2.46×10^4	2.51×10^4	3.21×10^4
16	11.79	lactic acid	117	1.83×10^5	1.35×10^5	9.55×10^4
17	12.14	citric acid	86	2.50×10^4	2.03×10^4	2.40×10^4
18	12.8	glucose	147	2.06×10^5	2.02×10^5	1.95×10^5
19	12.89	erythrose-4-phosphate	131	7.12×10^4	7.19×10^4	1.16×10^5

20	14.02	ribulose-5-phosphate	97	5.71×10^4	5.70×10^4	7.95×10^4
21	14.11	ribose-5-phosphate	236	3.10×10^4	2.80×10^4	1.56×10^4
22	15.36	fructose-6-phosphate	506	8.40×10^3	7.27×10^3	5.24×10^3
23	15.74	glucose-6-phosphate	204	3.09×10^3	2.80×10^3	1.78×10^3
